# Test–Retest Reliability and Preliminary Validation of Smartphone-Based Threshold and Categorical loudness Measures in Home Settings

**DOI:** 10.1177/23312165261471286

**Published:** 2026-08-20

**Authors:** Chen Xu, Lena Schell-Majoor, Birger Kollmeier

**Affiliations:** 1 Medizinische Physik and Cluster of Excellence Hearing4all, 597451 Universität Oldenburg, Oldenburg, Germany; 2 Institute of Sound and Vibration Research, Faculty of Engineering and Physical Sciences, University of Southampton, Southampton, UK

**Keywords:** remote audiology, ambient noise, validity and reliability, categorical loudness scaling

## Abstract

Reliable hearing assessment at home can improve accessibility and reduce dependence on in-clinic testing. To be viable, home-based procedures must provide accurate results within short measurement times and remain robust to factors such as ambient noise and variable user attention. In this proof-of-concept study with a limited sample size, we evaluated two such procedures—a Graded Response Bracketing method (GRaBr) for pure-tone threshold estimation and a reinforced adaptive categorical loudness scaling method (rACALOS) for loudness-growth assessment - using remote, smartphone-based testing, and compared their outcomes with those obtained inside the laboratory using established reference procedures. Fifteen young adults with normal hearing completed the tasks at home and in the laboratory. Test–retest reliability was assessed by repeating the home measurements within one week of the initial session. Ambient noise levels in home environments were also recorded. The intraclass correlation coefficients for GRaBr measured at home exceeded 0.75, indicating good test–retest reliability. Similarly, home-based rACALOS showed generally high reliability, with across-run biases below 5 dB at all frequencies; however, mean interquartile ranges reached up to 18.4 dB at some loudness categories. Remote GRaBr measurements showed small, non-significant deviations from laboratory audiometry (0.4 ± 7.1 dB); however, frequency-specific biases of approximately ±5 dB were observed at 250 Hz and 4 kHz, with root-mean-square errors ranging from 4.6 to 8.0 dB across frequencies. Compared with the in-lab ACALOS, remote rACALOS showed an overall bias of 3.38 ± 11.8 dB across loudness categories, with no significant overall effect of test environment. These findings suggest that smartphone-based pure-tone audiometry and loudness-scaling assessments provide accurate and reliable results when using these procedures at home, at least in normal-hearing young adults, who are experienced loudness raters, under suitable acoustic conditions with low ambient noise.

## Introduction

Pure-tone audiometry, a threshold-based testing approach, is widely used to detect hearing loss and guide audiogram-based hearing aid fitting. Its application for mobile and remote testing has therefore received much interest (see Almufarrij et al., 2022 for a review). In contrast, supra-threshold methods such as categorical loudness scaling (Hellbrück, 1987; Kollmeier, 1997) to characterize supra-threshold deficits and support loudness-based fitting have received much less attention in the literature on mobile or remote applications (Kopun et al., 2022; Xu et al., 2024a). However, this method appears to be particularly suitable for such applications because it is inherently robust to device calibration offsets (i.e., systematic differences between nominal and actual device output levels) (Almufarrij et al., 2022; Xu & Kollmeier, 2026). This robustness arises because the estimation of key parameters (e.g., dynamic range and the slopes of the loudness growth functions) relies on level differences rather than on absolute levels. Hence, both of these methods are attractive for mobile and remote testing if they are reliable, robust against disturbances, and provide valid estimates of what would be measured in a clinical setting. The current paper aims at evaluating refined versions of them that are designed to meet these requirements.

Accurate estimation of hearing thresholds remains essential for obtaining reliable reference data in both threshold- and supra-threshold-based tests. Previous work examined the effect of experimenter supervision on smartphone-based pure-tone audiometry and adaptive categorical loudness scaling (ACALOS, Brand & Hohmann, 2002) in a sound-attenuated booth with normal-hearing and hearing-impaired listeners, showing no significant supervision effect (Xu et al., 2024b). To further improve threshold estimation with respect to efficiency and robustness against inattention, we developed the graded adaptive response bracketing (GRaBr) method (Xu et al., 2024a), a model-free adaptive procedure that replaces the conventional yes-no paradigm with a two-interval task in which listeners indicate whether they heard none, one, or two sounds across the two intervals. Compared with the single-interval up–down (SIUD) method, which presents a cue tone in a first interval at a fixed level above the probe tone (Lecluyse & Meddis, 2009), GRaBr adaptively adjusts this level difference. Simulation studies, including Xu et al. (2024a), showed that this adaptive strategy yields substantially higher efficiency and robustness, particularly under both long- and short-term inattention. However, a preliminary validation of the remote GRaBr listening test with real participants in a home environment—where susceptibility to distractions is higher—against laboratory-based assessments is still lacking, which motivated a part of the present study.

Furthermore, we consider the ACALOS procedure (Brand & Hohmann, 2002) for remote testing, since it has been widely used as a practical and clinically relevant method to characterize loudness growth. In comparison to methods such as absolute magnitude estimation (Marks & Florentine, 2010), categorical loudness scaling uses more intuitive verbal response labels that assume loudness judgments are performed on an ordinal scale rather than on a ratio scale (Hellbrück, 1987). The Oldenburg variant (Kollmeier, 1997) increased reliability compared to the Allen et al. (1990) procedure by employing 5+2 verbal response labels (from “inaudible” via “very soft” to “very loud” and “too loud”), plus four intermediate, unlabeled response options, and a pseudo-random stimulus presentation order that avoids some unwanted sequence or range effects and ensures that previous experience with loudness scaling has little influence on the resulting judgments. Its adaptive version, known as ACALOS, optimizes the placement of stimulus levels by adaptively setting the upper and lower stimulus level range in an iterative procedure. This makes the procedure simple to perform for a variety of applications (e.g., Van Eeckhoutte et al., 2016).

However, despite its advantages for assessing supra-threshold hearing, the conventional ACALOS procedure often fails to accurately predict audiometric thresholds (Al-Salim et al., 2010; Oetting, 2014). Oetting et al. (2014) reported that the mean intra-subject standard deviation of loudness levels close to the threshold was notably high (around 10 dB) in comparison to much lower mean intra-subject standard deviations for levels corresponding to higher loudness labels (e.g., below 5dB for the “too loud” label). In addition, fluctuating ambient noise in home environments may pose challenges and could affect loudness judgments at low sound pressure levels (SPL). This all may contribute to the significant variability in the hearing threshold estimation from loudness judgments near the threshold. To address this, we propose here the reinforced adaptive categorical loudness scaling (rACALOS) method (Xu, 2025), which retains the stimulus type and the psychophysical paradigm and refines the ACALOS procedure by incorporating an improved threshold estimation process. This approach combines threshold and supra-threshold assessments in a single, time-efficient test, improving estimation accuracy of the hearing threshold and facilitating remote implementation on smartphones.

In the current study, we aim at evaluating the performance and test-retest reliability of these novel smartphone-based methods for remote hearing assessment - GRaBr for threshold estimation and rACALOS for categorical loudness scaling - when conducted by a limited number of young, normal listeners in home environments in comparison to the reference tests conducted inside the laboratory using the baseline procedures. We hypothesized that the smartphone-based procedures would achieve accuracy and reliability comparable to laboratory-based measurements and evaluated their performance under non–sound-treated home environments, a prerequisite for future integration into mobile hearing assessment platforms.

## Methods

### Participants

Fifteen young adults with normal hearing (aged 20 to 35 years; 4 men, 11 women) participated in this study. All participants were members of working groups or students at the University of Oldenburg and were recruited primarily through informal requests within the labs. The three authors did not participate in the study as subjects. All participants self-reported no hearing issues and had previously been classified as having normal hearing (NH) based on pure-tone audiograms using the SIUD procedure with thresholds within the normal-hearing range up to 8 kHz in earlier studies (Xu et al., 2024b). Two inclusion criteria were applied: (i) the air-conduction pure-tone average (PTA-4) at 0.5, 1, 2, and 4 kHz in the better ear had to be less than or equal to 20 dB HL, and (ii) symmetric hearing, defined as a threshold difference of no more than 20 dB between ears at any test frequency. All 15 participants met these criteria. Some listeners (N = 5) received compensation of €12 per hour for their participation, while others took part as part of their work duties. The study was approved by the Research Ethics Committee of the University of Oldenburg (Drs. EK/2023/004).

### Reference Laboratory-Based Testing Inside a Booth

We reused the data set from our previous study (Xu et al., 2024b) as the reference laboratory-based listening tests. Full methodological details are provided in Xu et al. (2024b). Following a repeated-measures design, the participants in the present study were the same individuals as those in Xu et al. (2024b). Therefore, they were already familiar with the testing procedures. The remote measurements were conducted more than three months after the laboratory session, which likely reduced potential learning effects.

All measurements were conducted separately for each ear by an experimenter inside a sound-attenuated booth. Hearing thresholds were obtained using the SIUD procedure, which has been shown to yield comparable mean results with higher reliability than clinical pure-tone audiometry using the Hughson–Westlake procedure (Lecluyse & Meddis, 2009). Loudness growth functions were assessed using the ACALOS procedure (Brand & Hohmann, 2002).

### Equipment, Procedure, and Environment for Remote Testing

Prior to the start of remote testing, a test kit was assembled, which included a smartphone (OnePlus, Android) and HD650 circumaural headphones (Sennheiser, Wedemark, Hanover, Germany). The smartphone and headphones were pre-calibrated with frequency-response equalization using a Brüel & Kjær (B&K) artificial ear 4153, a B&K 0.5-inch microphone 4134, a B&K microphone pre-amplifier 2669, and a B&K measuring amplifier 2610, with a target calibration level of 80 dB SPL. There was only one test kit in use, and the setup was calibrated once at the beginning of data collection. Upon handing over the test kit, participants received a brief oral explanation of the remote experiments, and consent forms were signed before they began. The oral instructions consisted of three parts: test environment check, internet connection check, and steps for conducting the remote experiments. The details are provided below. Participants could initiate testing at home by connecting to the internet via WLAN and accessing the provided website. For data security, a VPN connection was established using the “GlobalProtect” app when accessing the site. The VPN connection was required to allow external participants to access the university’s internal server hosting the listening tests and was implemented due to institutional IT security policies rather than as a requirement of the method itself. The workflow of the web-based application for remote testing was described in Xu et al. (2024b). A Raspberry Pi 3 Model B (Raspberry Pi Foundation, UK), a Linux-based micro-controller, served as the server hosting the measurement site. All behavioral data were stored on an SD card within the Raspberry Pi, located at the University of Oldenburg.

Listeners were asked to complete remote testing at home on two separate days within a week—one for the test measurement and the other for the retest measurement. Each day’s testing lasted approximately half an hour, resulting in a total testing time of one hour per participant. The home environments were primarily located in rural regions of northwestern Germany and cities such as Oldenburg, Cloppenburg, Jever, and Bad Zwischenahn.

### Noise Level Measurement for Remote Testing

Ambient noise was measured using the freely available “Decibel X” smartphone application (SkyPaw Co., Ltd.), which has been shown to provide reasonably reliable environmental sound level estimates on modern smartphones when compared with standard sound level meters (e.g., Scharf et al., 2024). We configured the app with an A-weighted filter and a 500-ms slow time weighting. The app displayed real-time, average, and maximum noise levels but did not record audio. To calibrate the smartphone’s internal microphone, a Class 3 digital sound level meter (Voltcraft SL-100; ±2 dB at 1 kHz), previously calibrated in our laboratory, was placed next to the phone. The smartphone microphone was positioned approximately 20 cm from the laptop speaker while an 80-dB SPL one-third-octave-band narrowband noise with a smooth envelope centered at 1 kHz was presented via the laptop speaker (Kohlrausch et al., 1997; Xu et al., 2024b). Because the app supports only a single calibration value, the offset measured at 1 kHz was used as the overall calibration offset, although calibration offsets may vary across frequencies. The app’s gain was then adjusted to match the digital sound level meter, resulting in a calibration offset of 13.7 dB that was added to the app readings. The same smartphone and headphones were used for all participants to ensure consistent gain.

At the beginning of each remote session, participants documented the current ambient noise level. They were instructed to monitor real-time noise using the app and to pause testing whenever levels exceeded 45 dBA, a threshold selected based on Kopun et al. (2022), who showed that remote loudness-scaling results remain comparable to laboratory measurements below 50 dBA ambient noise level. The time and location of each remote session were also recorded.

### Remote Listening Tests at Home

A total of 24 sessions were conducted by each participant at home, consisting of 4 listening tests (SIUD, GRaBr, ACALOS, and rACALOS; see details below) across 3 test frequencies (0.25, 1, and 4 kHz) and 2 runs (test and retest), presented in randomized order. Please note that the test and retest measurements are referred to as Run 1 and Run 2, respectively. Participants were instructed to take short breaks between sessions.

#### Remote Pure-tone Audiometry

Remote pure-tone thresholds were measured using two adaptive procedures: the SIUD method and the GRaBr approach (Lecluyse et al., 2009; Xu et al., 2024a). In both tasks, each trial contained a higher-level cue tone followed by a probe tone, and listeners reported whether they heard zero, one, or two tones. The cue tone was muted on 20% of trials (catch trials). The key difference between the procedures is that SIUD uses a fixed 10-dB level difference between probe and cue tones, whereas GRaBr adapts this difference: it starts at 10 dB, decreases to 5 dB after the first “one-tone” response, and to 2 dB after the second.

In SIUD, the step size of the probe tone level began at 10 dB and was reduced to 2 dB after the first “one-tone” response (by setting the level to the midpoint of the preceding two levels). In GRaBr, step sizes were 8, 6, 3, and finally 1 dB after the first, second, and third reversals, respectively. In both procedures, levels were adjusted adaptively—becoming harder after correct responses and easier after incorrect ones. To enable fair comparison, key parameters (e.g., minimum trials, reversals, starting level) were matched. The initial cue-tone level for both methods was drawn from a discrete uniform distribution of 55–65 dB SPL (1-dB steps). The adaptive track was terminated after at least 14 reversals were performed, and the first four reversals were discarded. Each pure tone lasted 0.2 s, with cosine ramps of 0.02 s and a 0.3 s interval between tones. In SIUD, the correct response rates were fitted to an S-shaped logistic psychometric function, and the level at the 50% correct response point (L_50_) was estimated as the hearing threshold. For GRaBr, responses from the upper and lower tracks (i.e., the tracks of the cue tones and the probe tones) were fitted to two independent psychometric functions, and the hearing threshold was calculated as the mean level at the 50% correct response point of both functions (i.e., 0.5*(L_50,upper_ + L_50,lower_)).

#### Remote Adaptive Categorical Loudness Scaling

**Figure 1.**
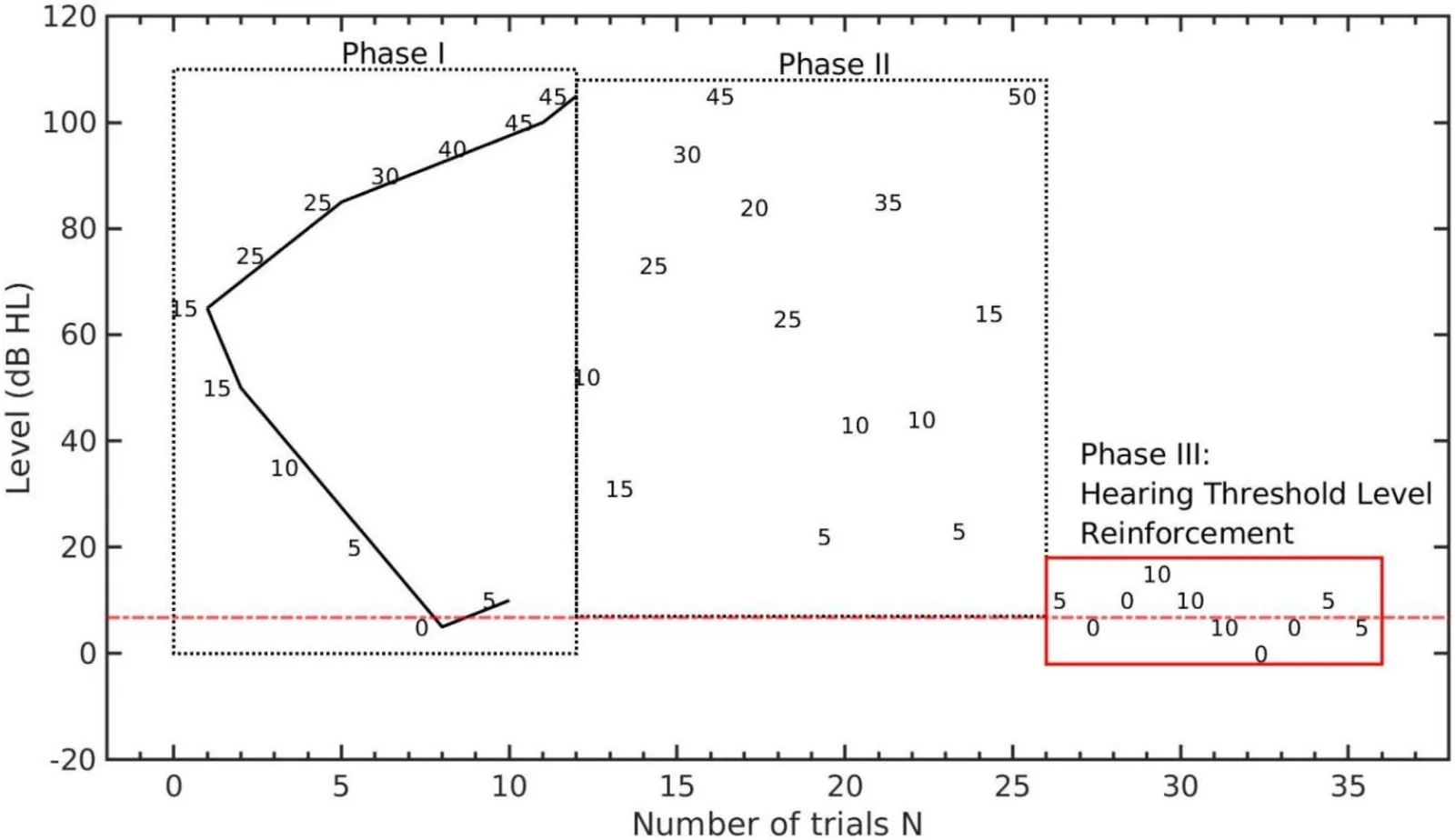
An example track of the reinforced adaptive categorical loudness scaling (rACALOS), where the level (in dB HL) is plotted as a function of the number of trials N. The listener’s response (in categorical units (CU)) is annotated with numbers between 0 (‘not heard’) and 50 (‘too loud’). Left dotted rectangle region: Phase I (‘dynamic range estimation’); Middle dotted rectangle region: Phase II (‘presenting and re-estimation’); Right solid red rectangle region: Phase III (‘hearing threshold level reinforcement’); Red dash-dotted line: target threshold. In Phase III, the step size is set to 5 dB, and the number of trials is set to 10

Both the ACALOS (Brand & Hohmann, 2002; ISO 16832, 2006) and the rACALOS method were used to assess the loudness growth functions. In the ACALOS task, participants rated the loudness of stimuli on an 11-point scale with descriptors ranging from “very soft”, “soft”, “medium”, “loud”, and “very loud” with 4 unnamed intermediate categories in between, plus the two limiting categories “not heard”, and “too loud”. The stimuli used were one-third-octave-band low-noise noises (i.e., narrowband noises with an optimized temporal envelope that minimizes temporal level fluctuations while preserving the same spectrum; Kohlrausch et al., 1997). Each noise stimulus had a duration of 1 second with 0.05-second rise and fall ramps. The stimulus levels, ranging from -10 to 105 dB, were presented in a pseudo-random order following an initial estimation of the user-specific dynamic range (Phase I, see Figure 1), which was updated to obtain a more representative placement of test level in Phase II, encompassing 26 trials. At the end of the procedure, a loudness growth function was modeled by fitting two linear segments and a transition region using a Bezier fit, following the BTUX fitting method (Oetting et al., 2014). Each frequency was assessed in a separate run.

An example run of the proposed rACALOS procedure is shown in Figure 1. The rACALOS followed the same adaptive rules as ACALOS during Phases I and II (see above) but presented additional stimuli near the hearing threshold to better estimate the hearing threshold level (HTL). The starting level of Phase III was set at the minimum level reached in Phases I and II, plus 5 dB. In this phase, a one-up-one-down adaptive rule was applied: the stimulus level increased by 5 dB if participants responded with “not heard” and decreased by 5 dB if they selected other loudness categories (e.g., “very soft,” “medium”). Phase III consisted of 10 trials. The fitting of the loudness functions from the rACALOS data was performed using all data points across phases.

### Data Analysis

Data from each test frequency were analyzed separately. Measurements were obtained monaurally, with 250 Hz and 4 kHz tested in the left ear and 1 kHz tested in the right ear. The same ear–frequency assignments were maintained across all test sessions and runs. Consequently, no averaging across ears was performed. For each run, the order of test frequencies (and thus the associated test ears) was randomized across participants to minimize potential order-related effects.

#### Test–Retest Reliability

The test–retest reliability of the GRaBr procedure in the home environment was primarily quantified using the intraclass correlation coefficient (ICC(2,k), two-way random-effects model, average measures). In contrast, the reliability of rACALOS was evaluated in terms of across-run bias and within-run variability, following Kopun et al. (2022).

#### Validity

The validity of the GRaBr procedure was assessed using Spearman’s correlation coefficient (R) between hearing thresholds measured remotely using GRaBr and reference hearing thresholds measured inside the laboratory using the SIUD procedure, as well as bias (defined as the mean signed difference across participants) and root-mean-square error (RMSE). For rACALOS, validity was likewise characterized using R, RMSE, and bias, reported as a function of CU.

## Results

The Results section is organized as follows. First, ambient noise measurements obtained during home testing are presented. Next, the test–retest reliability of the home-based audiometric and loudness-scaling procedures is examined. The validity of the remote procedures is then evaluated by comparison with corresponding laboratory-based reference measurements. Finally, hearing thresholds derived from loudness-scaling procedures are compared with those obtained using pure-tone audiometry.

### Noise Level Measurements

**Figure 2.**
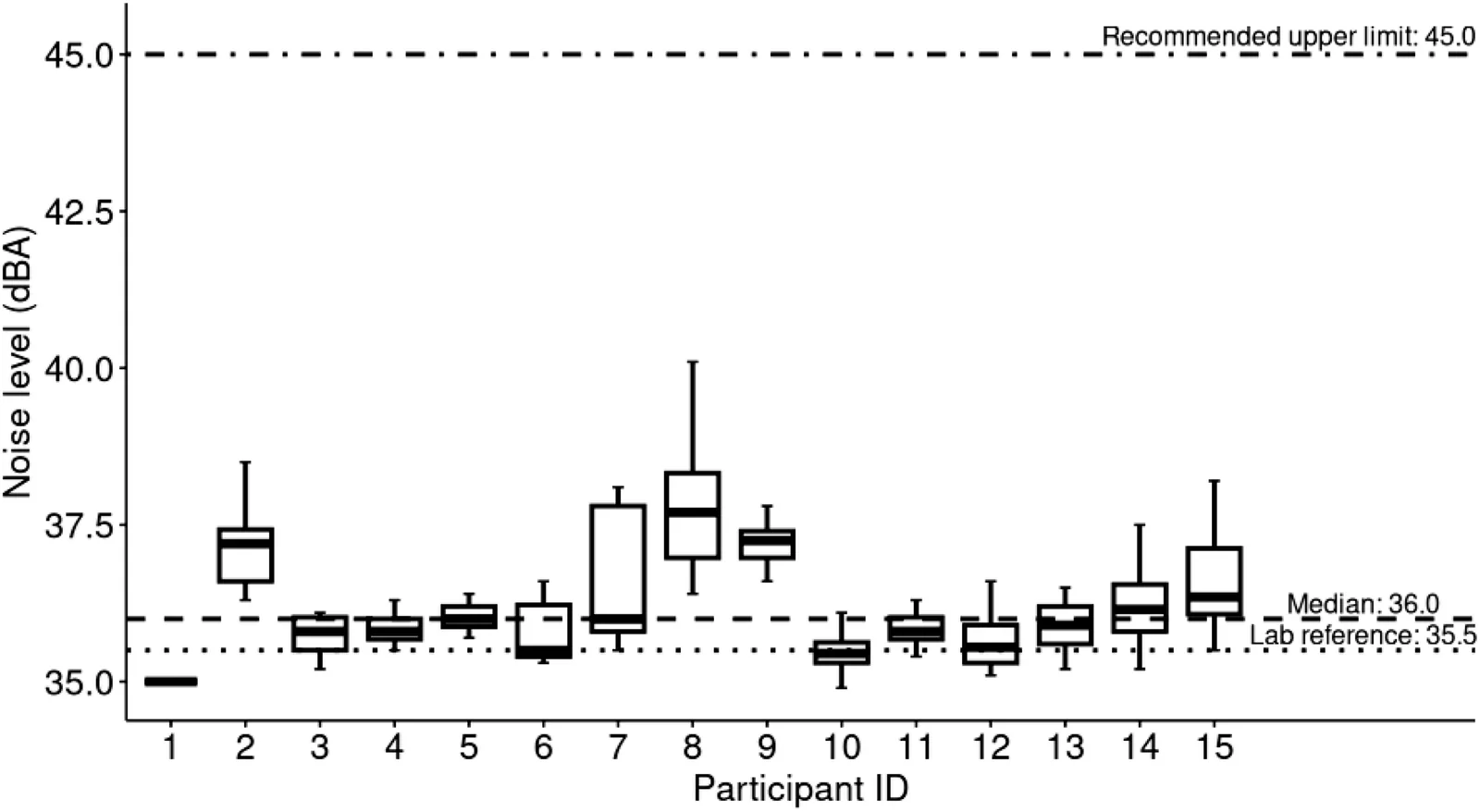
Ambient noise level (in dBA) measurement across participants (N = 15). Data are presented as boxplots showing the median and the interquartile range (IQR). Whiskers represent the range of observations excluding outliers (values exceeding 1.5×IQR). Dotted line: lab reference (i.e., ambient noise level measured within a booth). Dashed line: median value across subjects. Dot-dashed line: recommended upper limit

Ambient noise levels recorded by each participant (N = 15) are summarized in a box plot (Figure 2). Participants documented the ambient noise level 24 times at the start of each measurement session, corresponding to 24 home measurement sessions conducted within one week. Notably, the noise levels for all participants remained below the recommended upper limit of 45 dBA. The median noise level across subjects was 36.0 dBA, which was approximately 0.5 dB higher than the reference noise level measured inside the sound-attenuated booth. The interquartile range (IQR) across participants was 1.2 dB. Overall, the sound levels in participants’ homes were considerably low and comparable to those measured within the booth, indicating a suitable test environment. A few participants (e.g., No. 2 and No. 8) lived near a train station, resulting in slightly elevated noise levels compared to others. Additionally, one participant (No. 1) misinterpreted the task and consistently rounded the recorded noise level to an integer, resulting in all recorded values being 35 dBA across sessions.

### Test-Retest Reliability for Home Testing

**Figure A1.**
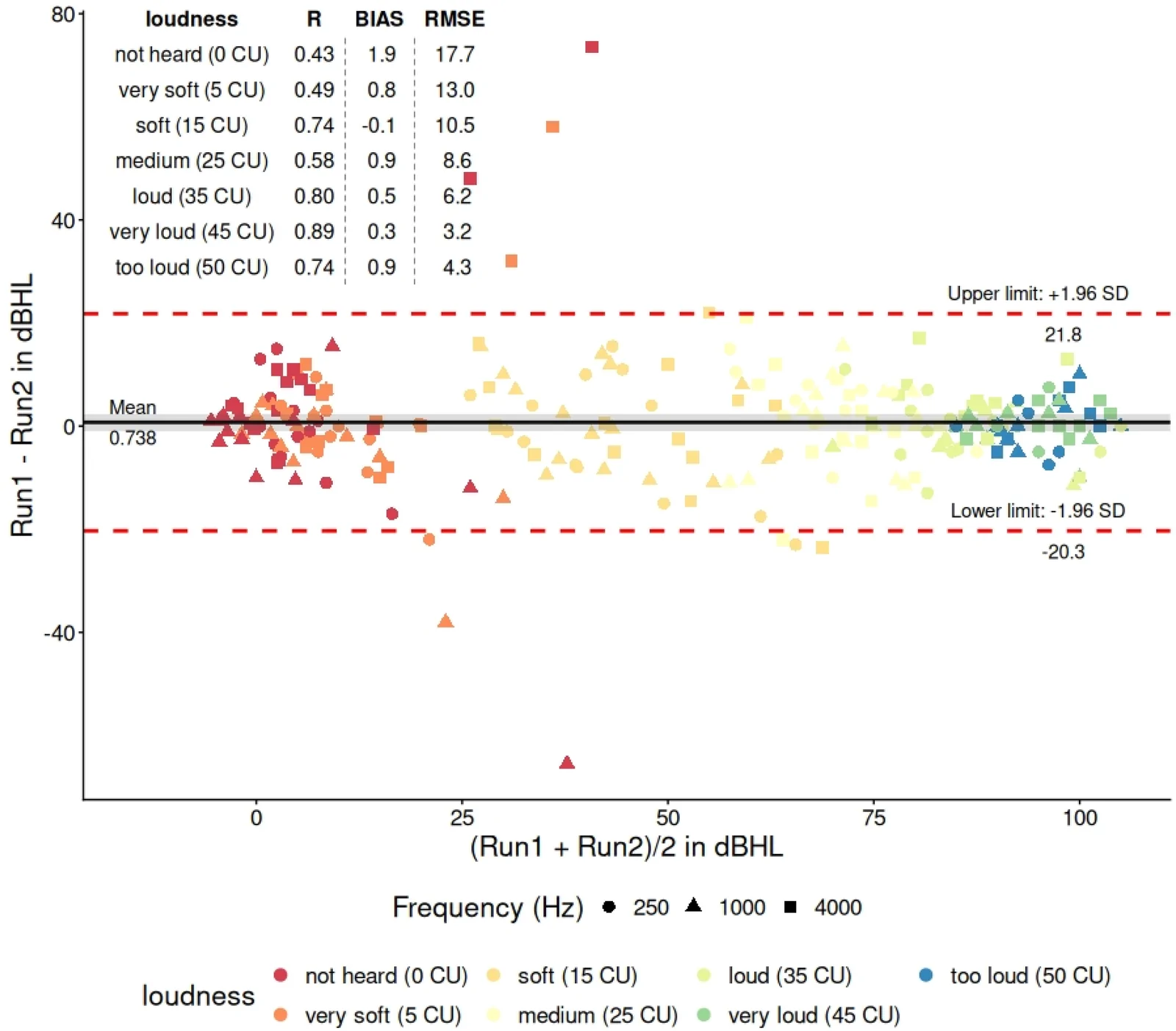
Bland-Altman plot assessing the reliability of **rACALOS** by comparing two runs in a home environment. Each point represents a loudness judgment. CUs are distinguished by color, while frequencies are represented by different shapes. R, BIAS, and RMSE as a function of CU are provided in the top-left corner of the figure. Red dashed lines: 95% limits of agreement; Black solid line: bias between the two runs; Grey shaded rectangle area: 95% confidence interval of the bias.

To assess the reliability of the four home-based listening tests, measurements from the initial run (Run 1) were compared with those from the retest (Run 2). Details of the statistical analyses in terms of reliability for remote ACALOS and rACALOS testing are provided in the supplementary material (see Table S2). An agreement analysis for rACALOS is presented in the Appendix (see Fig. A1).

#### SIUD and GRaBr for Home Testing

**Figure 3.**
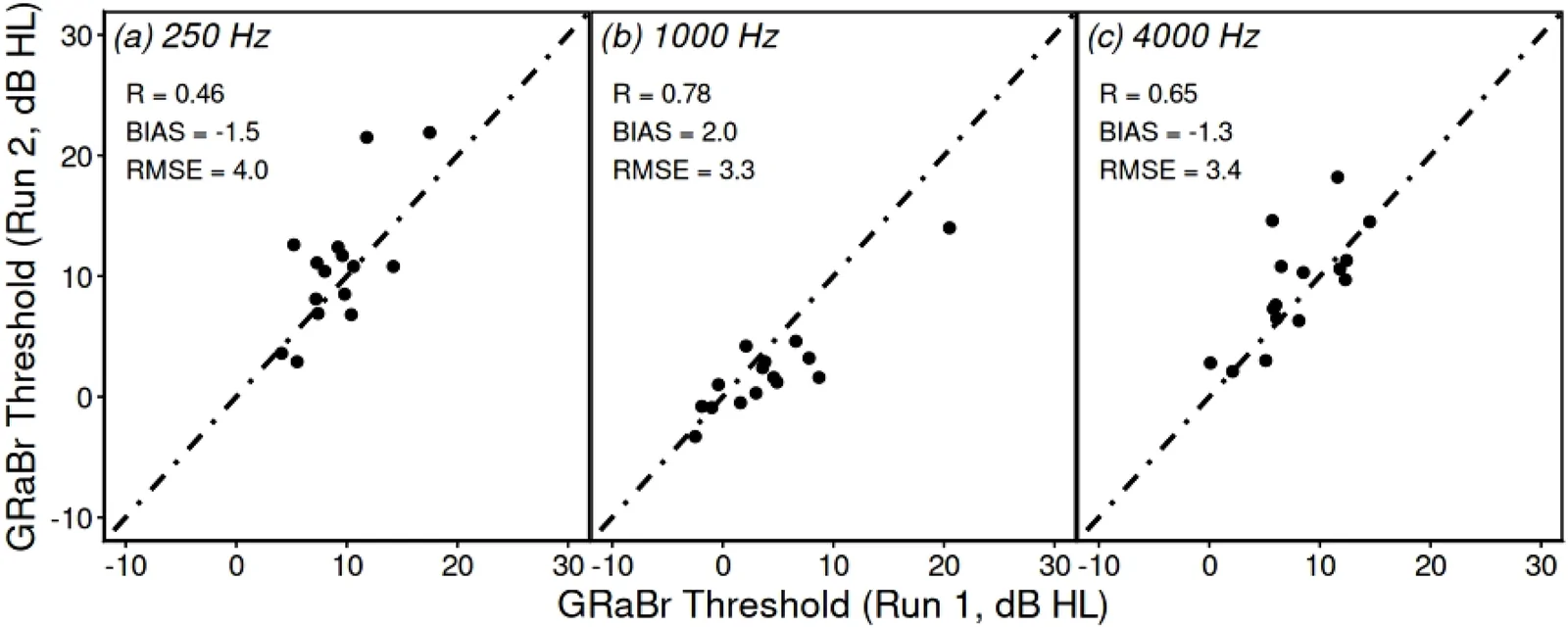
Hearing thresholds (dB HL) obtained in Run 2 are plotted against those from Run 1 for N = 15 participants using the GRaBr procedure in a home environment at 0.25, 1, and 4 kHz frequencies. Spearman correlation coefficients (R), bias (mean signed difference between the two runs), and root-mean-square error (RMSE) are shown in the top-left corner of each sub-figure. The dot–dashed line indicates the line of equality (y = x). Each dot represents one participant

Test–retest reliability of the GRaBr procedure was examined by comparing thresholds obtained in Run 1 and Run 2 (Figure 3), with results indicating high consistency across runs. As shown in Table 1, the GRaBr procedure showed test–retest intraclass correlation coefficients exceeding 0.75 (p = 0.00002), indicating good reliability across all three frequencies, whereas the SIUD procedure yielded ICC values ranging from 0.59 to 0.77 (p = 0.0052), reflecting moderate test-retest reliability. This difference was significant using a paired t-test (t (2) = 4.64, p = 0.043), i.e., GRaBr demonstrated significantly higher test-retest reliability than SIUD based on these metrics.

**Table 1.** Test-Retest Reliably Between Two Runs for Remote GRaBr and SIUD Testing at Three Frequencies. Correlation Coefficient (Both ‘Pearson’ and ‘Spearman’), RMSE, Bias, and Intraclass Cross-Correlation (ICC) Between the Test and Retest Measurements are Reported

​	GRaBr (N = 15)			SIUD (N = 15)		
Frequency (Hz)	250	1000	4000	250	1000	4000
R (Pearson)	0.70	0.90	0.73	0.43	0.61	0.63
R (Spearman)	0.47	0.78	0.65	0.40	0.57	0.65
RMSE	4.0	3.3	3.4	5.4	5.2	4.1
Bias	-1.5	2.0	-1.3	-1.7	-0.5	-1.4
ICC2k^a^	0.77	0.88	0.83	0.59	0.77	0.74

^a^ICC2k: Average random raters.

A three-way repeated-measures ANOVA on hearing thresholds, with run (Run 1 vs. Run 2), frequency (250, 1000, and 4000 Hz), and procedure (SIUD vs. GRaBr) as within-subject factors, revealed a significant main effect of frequency (F (2, 28) = 15.46, p = 0.00003). In addition, a significant interaction between frequency and procedure (F (2,28) = 3.83, p = 0.034) suggests that the differences between procedures varied across frequencies, with GRaBr yielding slightly higher thresholds at lower frequencies (approximately 1–2 dB at 250 and 1000 Hz), while no meaningful difference was observed at 4 kHz. Moreover, pairwise t-tests using Bonferroni correction were performed to assess reliability by comparing the two runs for both adaptive procedures across all three frequencies, showing no significant differences (bias) between runs in most cases, except for GRaBr at 1 kHz (t (14) = 2.85, p = 0.013).

#### ACALOS and rACALOS for Home Testing

**Figure 4.**
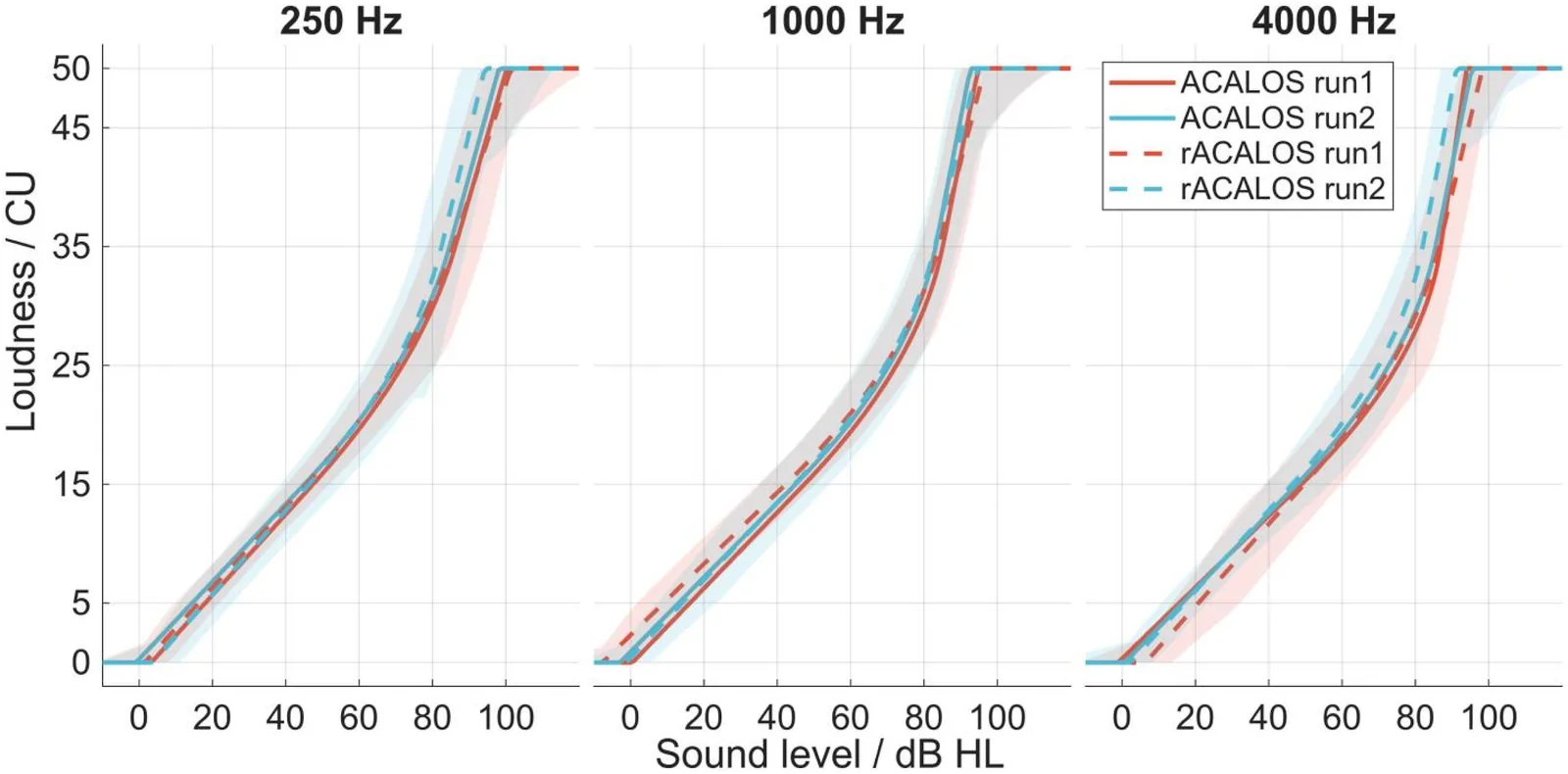
Mean loudness growth functions (loudness in categorical units as a function of sound level) for ACALOS and rACALOS measured at home, shown for two runs at three frequencies (N = 15 participants). Colors denote runs (red: Run 1; blue: Run 2), and line styles denote procedures (solid: ACALOS; dashed: rACALOS). Red and blue shaded regions represent ±1 standard deviation of the fitted loudness growth functions across participants for Run 1 and Run 2 of the rACALOS procedure, respectively

Average loudness growth functions across 15 participants for Run 1 and Run 2 are compared in Figure 4. Overall, the loudness functions obtained in Run 1 closely aligned with those from Run 2 for both ACALOS and rACALOS, indicating good test–retest reliability. A comparison between rACALOS and ACALOS suggests only minor differences, which appear to be more noticeable at lower loudness levels (CU < 15) at 1 and 4 kHz. The substantial overlap between the shaded regions indicates that the variability of the fitted loudness growth functions was comparable between Runs 1 and 2 of the rACALOS procedure (For the variability of the ACALOS loudness growth functions, see Appendix 2.).

To quantify these observations, the reliability of the ACALOS and rACALOS procedures was assessed using across-run bias, quantified by the mean signed difference (MSD; i.e., the average difference between two runs), and within-run variability, measured by the mean interquartile range (MIQR; i.e., the average spread between the 25th and 75th percentiles within a run). Both adaptive procedures demonstrated an MSD of less than 5 dB at all frequencies, indicating a small across-run bias. Most MIQR values did not exceed 10 dB for either procedure at the three frequencies, although they were typically larger than 10 dB at 5, 10, and 15 CU, reflecting a consistent within-run variability. Overall, these metrics suggested that both ACALOS and rACALOS exhibited strong reliability.

A linear mixed-effects model was fitted with categorical unit (CU; 0, 5, 10, … and 50 CU) as the dependent variable and stimulus level (dB HL), run, frequency, and procedure (ACALOS vs. rACALOS) as fixed effects, with participant included as a random intercept. The analysis revealed a significant main effect of procedure (F (1, 1736.0) = 10.13, p = 0.001), indicating a statistically significant difference between ACALOS and rACALOS. Compared to the ACALOS procedure, the rACALOS procedure yielded lower mean sound levels (by 1.9 dB) to achieve the same loudness category ratings. Since the rACALOS and ACALOS procedures are identical in Phases I and II, this difference is likely attributable to the additional trials included in Phase III of the rACALOS procedure (see Figure 1). The most comfortable loudness level (MCL) and uncomfortable loudness level (UCL) were comparable between the two procedures (see 25 and 50 CU in Figure 4), consistent with the absence of a significant stimulus level × procedure interaction (F (1,1736) = 2.87, p = 0.091).

No significant effect was found for frequency (F (2, 1736.1) = 2.22, p = 0.109), and no significant difference was observed between the two runs (test and retest measurements) (F (1, 1736.0) = 1.24, p = 0.266). A subsequent post-hoc comparison with Bonferroni correction compared sound levels between runs 1 and 2 at each frequency, indicating that sound levels for run 1 did not significantly differ from those for run 2 at 250 Hz (t (1736) = −1.54, p = 0.124) or 1 kHz (t (1736) = −0.39, p = 0.694). However, a significant difference was observed at 4 kHz (t (1736) = −2.45, p = 0.015). Nevertheless, the difference at 4 kHz between runs was small (≈ 1 dB), suggesting limited clinical significance despite statistical significance.

### Validation of the GRaBr Procedure in a Home Environment

**Figure 5.**
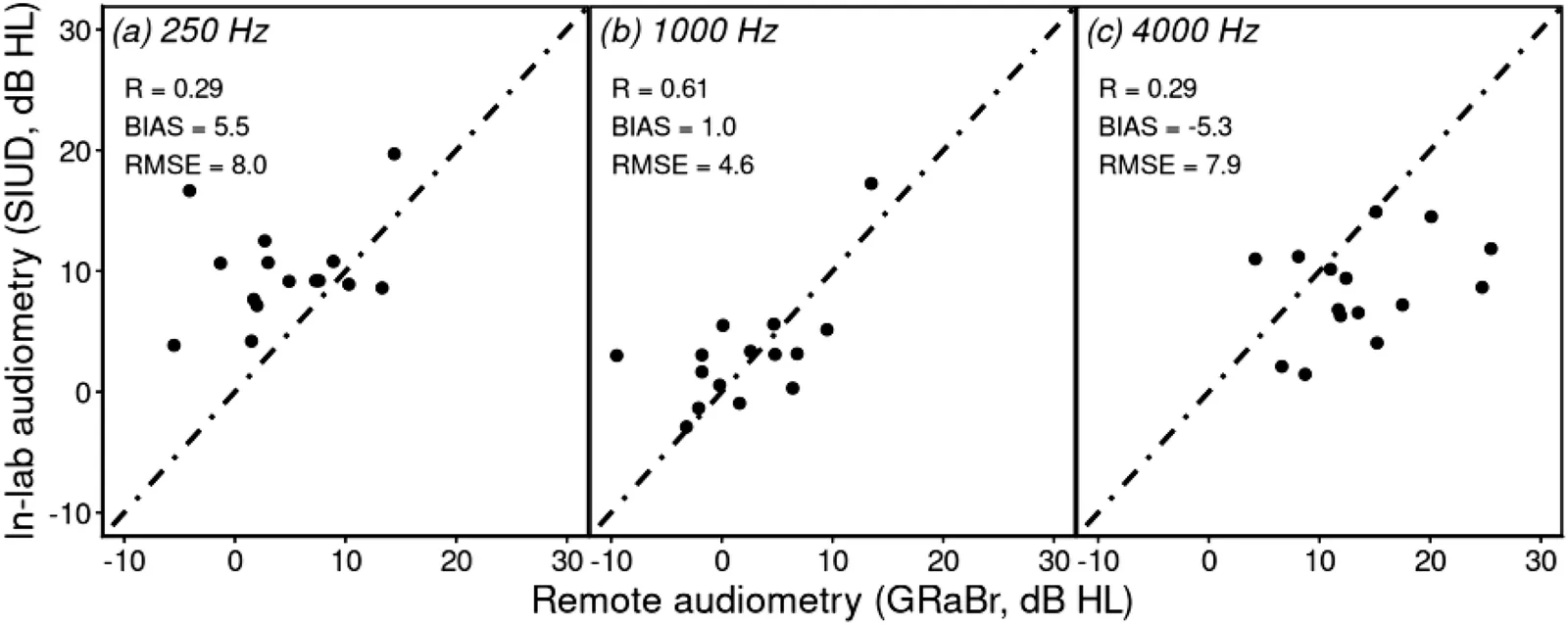
Comparison of hearing thresholds between in-lab audiometry (SIUD) and remote audiometry (GRaBr). The scatter plot layout and statistical annotations (top-left corner) are consistent with those presented in Fig. 3

To evaluate the home-based GRaBr procedure (remote audiometry), thresholds averaged across the two GRaBr runs were compared with booth-based thresholds obtained using the SIUD method (in-lab audiometry) at 0.25, 1, and 4 kHz, shown in Figure 5. Overall, some discrepancies were observed between the two measurements, indicating that agreement was not perfect.

However, the threshold differences were normally distributed (Shapiro–Wilk p = 0.92) and exhibited a small overall mean bias (mean signed difference across participants) of 0.4 ± 7.1 dB. Frequency-specific biases of approximately ± 5 dB were observed at 250 and 4 kHz that are larger than the biases between GRaBr and SIUD for home measurements reported above (approximately 1–2 dB at 250 and 1000 Hz). However, these values are within the typical 5-dB resolution used in clinical audiometry. The correlation between home and booth measurements was moderate—likely reflecting the limited variability in this normal-hearing sample—and the RMSE ranged from 4.6 to 8.0 dB across frequencies (7.0 dB overall), consistent with previous smartphone-based audiometry studies. Taken together, these results indicate that the GRaBr procedure for home testing provides broadly comparable threshold estimates to the reference in-lab measurements, supporting its overall validity.

A two-way repeated-measures ANOVA on thresholds, with test environment (lab vs. remote) and frequency (250, 1000, 4000 Hz) as within-subject factors, showed no main effect of test environment (F (1,14) = 0.09, p = 0.77). Frequency had a significant effect (F (2,28) = 20.80, p = 2.91 × 10^-6^), as did the environment × frequency interaction (F (2,28) = 36.01, p = 1.81 × 10^-8^), thus confirming the bias of approximately ± 5 dB at 250 and 4 kHz (see above). Post-hoc tests using Bonferroni correction revealed no significant difference between home and booth thresholds at 1 kHz (home: 3.1 ± 4.7, booth: 2.1 ± 5.7), while up to 5 dB significant differences were found at 0.25 kHz (home: 9.9 ± 4.1, booth: 4.4 ± 5.9) and 4 kHz (home: 8.4 ± 4.0, booth: 13.7 ± 6.2).

### Validation of the rACALOS Procedure in a Home Environment

**Figure 6.**
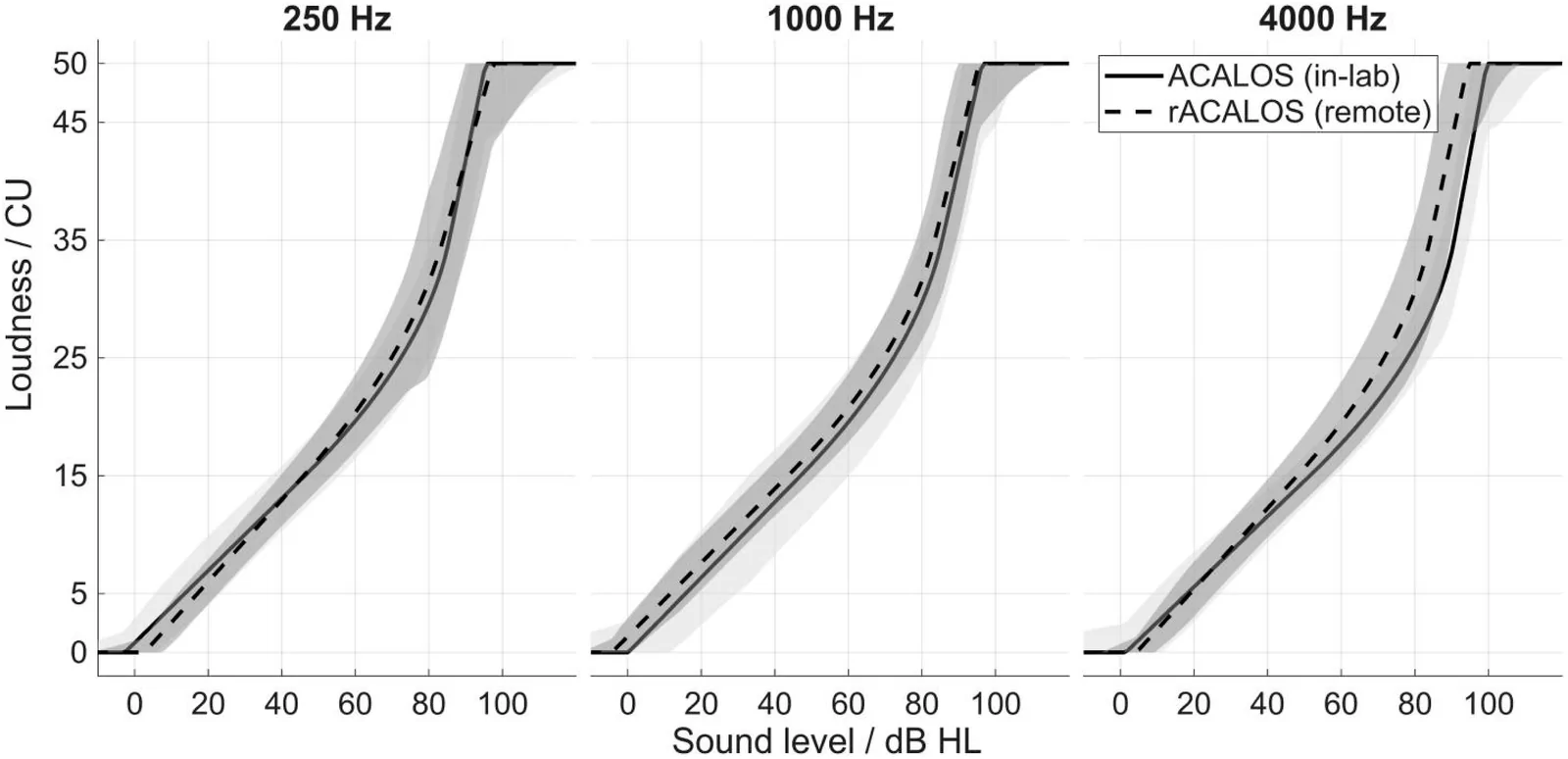
Comparison of average loudness growth functions between in-lab ACALOS and remote rACALOS. Light and dark gray shaded regions indicate ±1 standard deviation for in-lab ACALOS and remote rACALOS, respectively. The loudness curves follow the format described in Fig. 4

Loudness growth functions measured remotely at home using rACALOS were averaged across the two runs and compared with those obtained in the booth using the standard ACALOS procedure (i.e., in-lab ACALOS) at 0.25, 1, and 4 kHz (Figure 6). Overall, the average loudness growth functions showed good agreement between in-lab ACALOS and remote rACALOS, indicating good validity. Moreover, the considerable overlap between the shaded regions indicates similar variability in the fitted loudness growth functions obtained using the in-lab ACALOS and remote rACALOS procedures.

**Figure A2.**
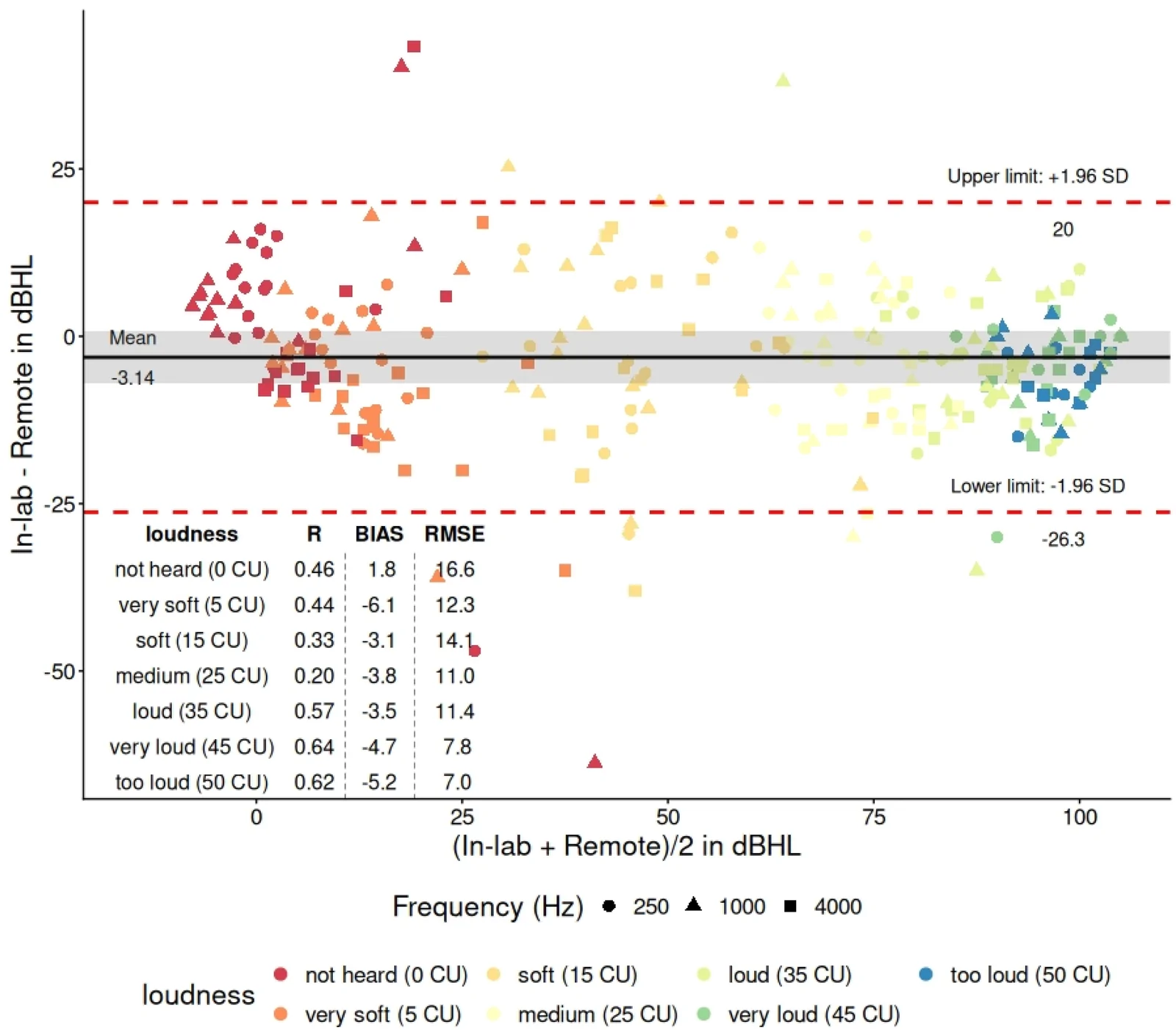
Bland-Altman plot assessing the validity of the remote rACALOS against the in-lab ACALOS. A comprehensive set of statistical measures containing R, Bias, and RMSE as a function of CU is provided in the embedded table located at the bottom-left corner. See Figure A1 for an explanation of the Bland-Altman plot.

Differences followed a normal distribution (Shapiro–Wilk p = 0.22), and the overall bias across CU was small (3.38 ± 11.8 dB), suggesting good validity of rACALOS relative to standard ACALOS. Agreement metrics varied across loudness levels: correlations for CUs ≥ 35 ranged from 0.57 to 0.62, whereas correlations for softer CUs were lower (< 0.45), accompanied by larger RMSE values. This pattern reflects the higher variability inherent at near-threshold levels rather than a systematic effect of home-environment noise, as deviations occurred in both directions. See Table S1 in the supplementary material for the details of the validation for the home-based rACALOS procedure and Appendix for the agreement analysis (see Fig. A2).

A linear mixed-effects model was fitted with CU as the dependent variable and stimulus level (dB HL), test environment, and frequency as fixed effects, with participant included as a random intercept. The analysis revealed a significant main effect of stimulus level (F (1, 892.53) = 7775.85, p < 0.001), indicating that loudness judgments increased systematically with increasing stimulus level. A significant main effect of frequency was also observed (F (2, 891.17) = 10.38, p < 0.001), whereas no significant main effect of test environment was found (F (1, 891.65) = 0.03, p = 0.87). A significant condition × frequency interaction was observed (F (2, 891.00) = 3.50, p = 0.031), indicating that environment-related effects varied across frequencies. Bonferroni-corrected post-hoc comparisons showed significant differences between laboratory and remote testing at 250 Hz (p = 0.043) and 4 kHz (p < 0.0001), whereas no significant difference was observed at 1 kHz (p = 0.096).

### Accuracy of HTL Estimation for the rACALOS Procedure

**Figure 7.**
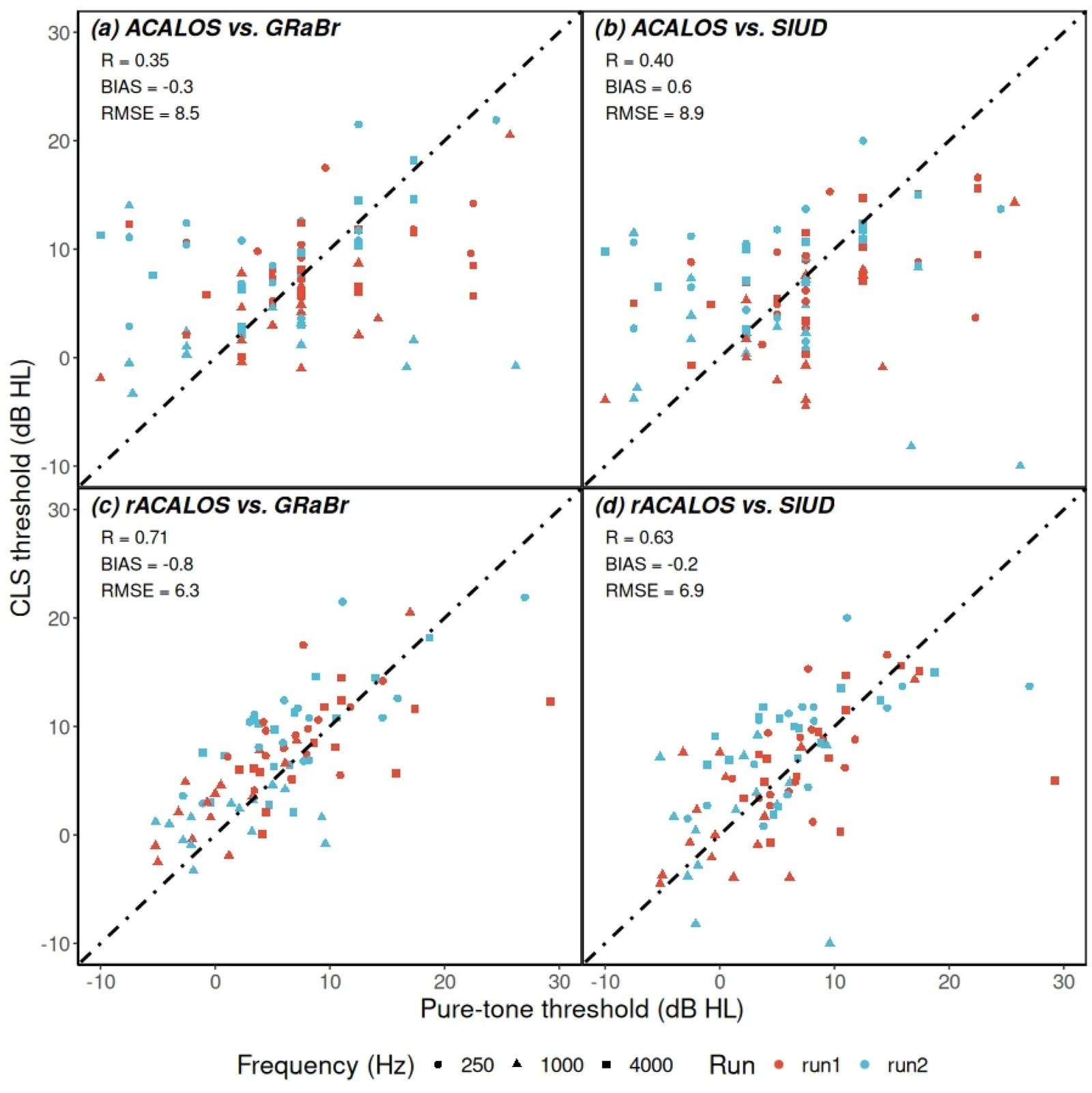
Scatter plot comparing pure-tone thresholds (x-axis) and CLS-derived thresholds (y-axis), both measured in the home environment. CLS thresholds are defined as the stimulus level at 2.5 categorical units (CU) of the loudness growth function, expressed in dB HL for N = 15 listeners. Frequency is labeled with different shapes while the run is denoted with different colors (Run1: red, Run2: blue). A set of statistical metrics (R, Bias, and RMSE) are reported in the top-left corner. For rACALOS, 10 additional trials with a step size of 5 dB were used

Pure-tone audiometric thresholds are plotted against CLS-derived thresholds (i.e., levels at 2.5 CU of the loudness growth functions) for two runs and three frequencies in Figure 7. Compared to ACALOS, the majority of rACALOS points were consistently and closely clustered around the diagonal line, indicating that thresholds estimated by the rACALOS method aligned more closely with pure-tone thresholds than those from baseline ACALOS and, hence, provide improved accuracy in threshold estimation. Quantitatively, R values increased by 36% for GRaBr and 23% for SIUD when ACALOS was reinforced near the hearing threshold level. Additionally, RMSE values for the rACALOS method decreased by approximately 2 dB compared to the baseline, while biases remained unchanged. Overall, the reinforcement of baseline ACALOS positively influenced cross-correlation and reduced error.

The highest correlation coefficient and lowest RMSE were observed between GRaBr thresholds and rACALOS, followed by SIUD and rACALOS. In contrast, the unmodified ACALOS procedure showed lower correlation coefficients and higher RMSEs for both threshold estimation methods, indicating the superior performance of rACALOS, as confirmed by t-tests.

## Discussion

This study examined the performance of two novel smartphone-based procedures—GRaBr for pure-tone audiometry and rACALOS for categorical loudness scaling—conducted by listeners independently in home environments. In this proof-of-concept study with a limited sample of normal-hearing participants, the results indicate that accurate and reliable measurements were obtained in our cohort without professional supervision or sound-treated booths. These findings provide evidence for the feasibility of remote hearing assessments using standard smartphones, although further validation in larger and more diverse populations is required.

### Ambient Sound Levels in Home Settings

The median background level measured across participants’ homes was 36.0 dBA, comparable to quiet residential environments and well below limits defined by ANSI S3.1–1999 (R2018) American National Standards Institute, 2018 for covered-ear conditions. These values fall within the updated permissible ranges for circumaural earphones proposed by Margolis et al. (2022), ensuring that ambient sound did not measurably affect the results. While noise levels were not experimentally manipulated, participants were advised to continuously monitor them to confirm that real-world domestic conditions remained suitable for valid measurements. Consequently, the obtained home results were closely aligned with those from the booth.

The recorded noise levels were approximately 4–10 dB lower than those in earlier remote audiometry studies (Brennan-Jones et al., 2016; Storey et al., 2014; Swanepoel et al., 2015) and comparable to the quietest non-sound-treated settings reported by Serpanos et al. (2022) and Bean et al. (2022). The smartphone-based listening tests were conducted by participants at their homes, predominantly in rural areas, with most measurements taking place during the morning or evening hours, whereas other studies typically test in clinical settings located in urban areas during the daytime, which tend to be noisier. Overall, the measured noise conditions indicate that sufficiently quiet environments for mobile hearing assessments can be achieved at least in some home settings.

### Pure-Tone Audiometry (GRaBr)

Smartphone-based audiometry with GRaBr showed good agreement with in-booth reference measurements, with a mean bias of 0.4 dB. While both SIUD (0.59 < ICC < 0.77) and GRaBr performed reliably, GRaBr achieved higher test–retest consistency (ICC > 0.75) and lower within-subject variability, suggesting improved stability, as predicted by simulation (Xu et al., 2024a). These findings extend previous validations of audiometry conducted outside a sound-attenuated booth (Maclennan-Smith et al., 2013; Serpanos et al., 2022; Swanepoel et al., 2015) to an unsupervised, mobile context.

The correlation between at-home and in-booth thresholds (R = 0.47) was lower than in studies involving hearing-impaired listeners (R > 0.9; Maclennan-Smith et al., 2013), likely because our normal-hearing sample exhibited limited threshold variability. Future refinements - such as automated quality control or remote calibration - may further optimize the GRaBr for remote threshold testing.

### (Reinforced) Adaptive Categorical Loudness Scaling

Both ACALOS and its reinforced version, rACALOS, yielded valid and reliable loudness functions in home conditions. The bias between in-booth and at-home measurements (3.4 dB) was about 2 dB smaller than that reported by Kopun et al. (2022), indicating strong agreement across environments. The enhanced rACALOS design produced smaller within-run (IQR) and across-run (MSD) variability than both baseline ACALOS and previously published categorical loudness scaling methods (Fultz et al., 2020; Kopun et al., 2022; Rasetshwane et al., 2015). For normal-hearing participants, the mean IQR values at 5 CU for rACALOS (7.7 dB at 1 kHz and 6.7 dB at 4 kHz) were notably lower than those in earlier studies (≈ 10–15 dB), reflecting improved stability near the hearing threshold.

Across-run bias was also smaller than in prior work, likely due to the lower average ambient noise level and the reinforced threshold estimation sequence. The additional trials near threshold in rACALOS substantially reduced estimation uncertainty, resulting in the smallest overall variability among tested methods. Compared with the performance metrics reported for alternative adaptive CLS frameworks in Table 5 of Fultz et al. (2020), rACALOS appears to provide higher precision and efficiency, supporting its use as a robust supra-threshold measurement for mobile and unsupervised hearing assessments.

### Accuracy of HTL Estimation

Table 2 presents a comparison between our current study and several state-of-the-art works (Fultz et al., 2020; Sanchez-Lopez et al., 2021; Trevino et al., 2016) by evaluating the cross-correlation between CLS and pure-tone thresholds. Multiple categorical loudness scaling methods, including Fixed-level (FL), Maximum expected information-Median (MEL-Med), Maximum expected information-Maximum likelihood (MEL-ML), Slope-adaptive (SA), ACALOS, and rACALOS, were used to estimate thresholds, which were then compared with pure-tone thresholds measured using various audiometric methods such as a clinical audiometer, SIUD, and GRaBr. In the studies by Fultz et al. (2020) and Trevino et al. (2016), Spearman correlation coefficients ranged from 0.21 to 0.26 for the threshold estimated from all four categorical loudness scaling methods, indicating a relatively weak cross-correlation. Additionally, the RMSEs and biases in these studies were notably large, suggesting that CLS thresholds did not align well with pure-tone thresholds. In contrast, Sanchez-Lopez et al. (2021) applied a baseline ACALOS method using the same audiometric procedure as Fultz et al. (2020), and while the R-value did not significantly improve, both RMSE and bias were notably reduced. In our study, we employed SIUD and GRaBr to measure pure-tone thresholds, yielding a stronger cross-correlation and smaller bias, although the RMSE was slightly larger or comparable to that reported by Sanchez-Lopez et al. (2021).

**Table 2.** Comparison of Threshold Estimates Obtained From Different Pure-tone Audiometry Methods and Categorical Loudness Scaling (CLS) Methods Across Several State-Of-The-Art Studies and the Present Study. Spearman Correlation Coefficients (R), Root-Mean-Square Error (RMSE), Bias, and Sample Size (N) are Reported for Each Method Combination. The Highest R Value and the Lowest RMSE and Bias Within Each Comparison Set are Highlighted in Bold. (FL = Fixed-Level; MEL-Med = Maximum-Expected-Information, Median; MEL-ML = Maximum-Expected-Information, Maximum Likelihood; SA = Slope-Adaptive, as Defined in Fultz et al. (2020))

​	Audiometric method	CLS method	N	R (spearman)	RMSE (dB)	Bias (dB)
Fultz et al., (2020); Trevino et al., (2016)	Audiometer	FL	17	0.21	12.2	-6.9
		MEL-Med		0.26	25.3	-18.0
		MEL-ML		0.26	15.5	-10.6
		SA		0.21	15.7	-8.4
Sanchez-Lopez et al., (2021)	Audiometer	ACALOS	11	0.24	7.1	-2.3
current	SIUD	ACALOS	15	0.44	9.4	1.5
	GRaBr			0.38	9.0	1.0
	SIUD	rACALOS		0.59	7.8	0.5
	GRaBr			**0.71**	**6.9**	**0.04**

Considering all the studies, the rACALOS method consistently produces thresholds closest to pure-tone thresholds, outperforming other categorical loudness scaling methods. This finding is consistent with computer simulations that examined threshold variability across different parameter combinations of rACALOS compared to the original ACALOS procedure. However, it is important to note that rACALOS requires more measurement time due to the increased number of trials focused on converging near the HTL. Based on representative recordings, the measurement duration was on the order of ∼1.5–2 minutes for ACALOS and ∼2–2.5 minutes for rACALOS, indicating a moderate increase in test time in practical settings. Additionally, while pure-tone thresholds obtained using clinical audiometers are still widely regarded as the “gold standard”, more precise (see Lecluyse & Meddis., 2009; Xu et al., 2024a) and criterion-free methods such as SIUD and GRaBr may produce stronger correlations with CLS thresholds. It is also crucial to recognize that this comparison is based on a small sample of young NH listeners, and the conclusions may differ if HI listeners are included or if a larger participant pool is studied. This consideration is particularly relevant for potential discrepancies between the narrowband noise thresholds estimated by the categorical loudness scaling methods used here and the pulsed pure-tone thresholds assessed via audiograms. While threshold differences in our study sample of young NH listeners were minimal, variations in stimulus characteristics—such as spectral extent and modulation spectrum—may yield threshold differences in naïve listeners with hearing impairments. Nonetheless, we also expect these differences to be minimal, as the low-noise, third-octave-band noise utilized here shares key perceptual characteristics with a frequency-modulated sinusoid with minor envelope fluctuations and an instantaneous frequency confined well within a critical band (cf. Zwicker & Fastl, 2013).

### Advantages of rACALOS

Compared with the original ACALOS procedure, rACALOS offers several potential advantages. First, by combining pure-tone audiometry and categorical loudness scaling within a single measurement protocol, rACALOS may improve time efficiency by eliminating the need for separate tests, instructions, and training procedures. In addition, rACALOS incorporates additional presentations near the hearing threshold level (HTL), which can improve the precision of HTL estimation (see Table 2). These modifications enable audiometric threshold estimation to be integrated directly into the loudness-scaling framework. Furthermore, rACALOS retains the same user interface and response paradigm as ACALOS, minimizing additional training requirements. As a result, participants familiar with ACALOS can readily use rACALOS, while new users need to learn only a single, consistent interface for both threshold and loudness assessments.

### Limitations and Outlook

In this study, we conducted smartphone-based listening tests outside of a sound booth, preceded by ambient noise level measurements. Given the recommendation for conducting tests in rather quiet acoustical conditions, the testing environments generally exhibited a low background noise level. However, many individuals live in urban regions with significant vehicle or industrial noise, where real-world environments are typically much noisier. Testing in such noisy conditions warrants further investigation. Potential solutions, such as circumaural muffs or noise-canceling earphones (NCE), could prove effective. For instance, Saliba et al. (2017) evaluated mobile-based audiometry under 50 dBA background noise, using passive and active noise cancellation by placing circumaural muffs over insert headphones, successfully reducing noise. Similarly, Clark et al. (2017) tested NCE (BoseQuietComfort 15) in a patient consultation room and found that NCE sufficiently attenuated ambient noise below the ANSI standards.

Another key concern for out-of-booth audiometric tests is distraction. As noted by Margolis et al. (2022), background noise not only causes direct masking but also acts as a source of distraction. Their study demonstrated that increasing background noise levels led to elevated hearing thresholds and higher subjective ratings of distraction. Similarly, Xu et al. (2024a) characterized distraction arising from internal noise as long-term inattention. They also proposed and simulated short-term inattention—where listeners are distracted by external events—during mobile hearing tests, though this has yet to be validated with human participants.

Future work should expand the sample size by including more normal-hearing (NH) participants and incorporating hearing-impaired (HI) participants. The present proof-of-concept study included a relatively small sample of NH listeners (N = 15), and therefore estimates of reliability and correlations should be interpreted with caution, as such estimates may vary across samples. Compared to NH listeners, we expect the validity of pure-tone audiometry and ACALOS tests in HI listeners to be comparable, as supported by previous studies (e.g., Bean et al., 2022; Hazan et al., 2022; Xu et al., 2024b), suggesting that hearing loss does not significantly affect test validity. Regarding reliability, HI listeners are expected to show similar or sometimes even higher test-retest reliability in audiometry than NH listeners (Hazan et al., 2022), likely due to their elevated hearing thresholds, which may reduce the impact of ambient noise. Similarly, in the ACALOS procedure, HI listeners may exhibit comparable or even greater reliability at lower levels of the loudness growth function (Kopun et al., 2022; Rasetshwane et al., 2015), although the background noise levels in the present study were very low. This may have a limited impact on the accuracy of the loudness growth slope fitted to the data, which is generally increased in hearing-impaired listeners exhibiting recruitment. However, since the slope estimate is derived from loudness judgments across multiple supra-threshold levels, it is considered a reliable and robust measure, even when obtained through self-administered smartphone assessments.

Another limitation of this study is the use of an integrated microphone for noise measurement. Studies like Kopun et al. (2022) recommend using an external microphone, such as the MicW iBoundary, which provides higher accuracy in capturing frequency characteristics and calibration precision compared to the integrated microphone used here.

Enhanced calibration of smartphone microphones can, in principle, be achieved using an external reference sound, such as a whistle tone produced by a standard empty beer bottle (Scharf et al., 2024). However, accurate calibration of the playback level remains a challenge and is essential for precise auditory measurements (Xu et al., 2026; Xu & Kollmeier, 2026). While pragmatic approaches such as biological calibration—using a normal-hearing reference listener to define 0 dB HL (Honeth, 2010; Masalski et al., 2014)—may offer a practical alternative, this aspect was beyond the scope of the present proof-of-concept study with its limited sample size. Future work could focus on data-driven estimation of calibration coefficients to enable fully unsupervised field applications (Xu & Kollmeier, 2026).

Finally, Shen et al. (2018), Shen et al., 2024, and Shen et al., 2026 introduced a quick categorical loudness scaling (qCLS) procedure based on a Bayesian adaptive method, which can estimate equal loudness contours within just 5 minutes. Given its efficiency and accuracy, incorporating qCLS into future smartphone-based loudness tests is worth considering. However, it remains uncertain whether qCLS can estimate hearing thresholds as precisely as the rACALOS developed in this study, highlighting the need for further research to evaluate its threshold accuracy in comparison.

## Conclusion

This study evaluates two smartphone-based methods for hearing assessment—graded response bracketing (GRaBr) for pure-tone threshold estimation and reinforced adaptive categorical loudness scaling (rACALOS) introduced here for categorical loudness scaling—under home conditions but with calibrated hardware. The findings suggest that both measures (that have shown their high efficiency and robustness in previous simulation studies) can provide reliable outcomes comparable to those obtained in controlled laboratory settings.

In the validation experiment, no significant overall differences between home and sound-attenuated environments were observed for pure-tone audiometry using GRaBr and categorical loudness scaling using rACALOS at 0.25, 1, and 4 kHz, suggesting that remote testing may provide comparable results. Test–retest results further indicate moderate-to-good reliability across sessions, supporting the consistency of home-based measurements.

GRaBr showed higher reliability and accuracy than the SIUD method across the tested frequencies, suggesting that it may be a promising approach for mobile threshold estimation, even though a small, frequency-specific bias was detected across both methods. Similarly, rACALOS produced threshold estimates closer to audiometric thresholds measured by SIUD and GRaBr than those obtained with the baseline ACALOS procedure, indicating its potential for improving hearing threshold estimation and test efficiency by combining threshold and loudness assessments.

Overall, the results support the feasibility of using GRaBr and rACALOS for reliable and efficient hearing assessments in real-world home environments, representing an initial step toward smartphone-based audiometry for large-scale or remote applications.

## Supplemental Material

Supplemental Material - Test–Retest Reliability and Preliminary Validation of Smartphone-Based Threshold and Categorical loudness Measures in Home Settings Supplemental Material for Test–Retest Reliability and Preliminary Validation of Smartphone-Based Threshold and Categorical loudness Measures in Home Settings by Chen Xu, Lena Schell-Majoor and Birger Kollmeier in Trends in Hearing.

Supplemental Material - Test–Retest Reliability and Preliminary Validation of Smartphone-Based Threshold and Categorical loudness Measures in Home Settings Supplemental Material for Test–Retest Reliability and Preliminary Validation of Smartphone-Based Threshold and Categorical loudness Measures in Home Settings by Chen Xu, Lena Schell-Majoor and Birger Kollmeier in Trends in Hearing.

## Figures and Tables

**Figure S3.**
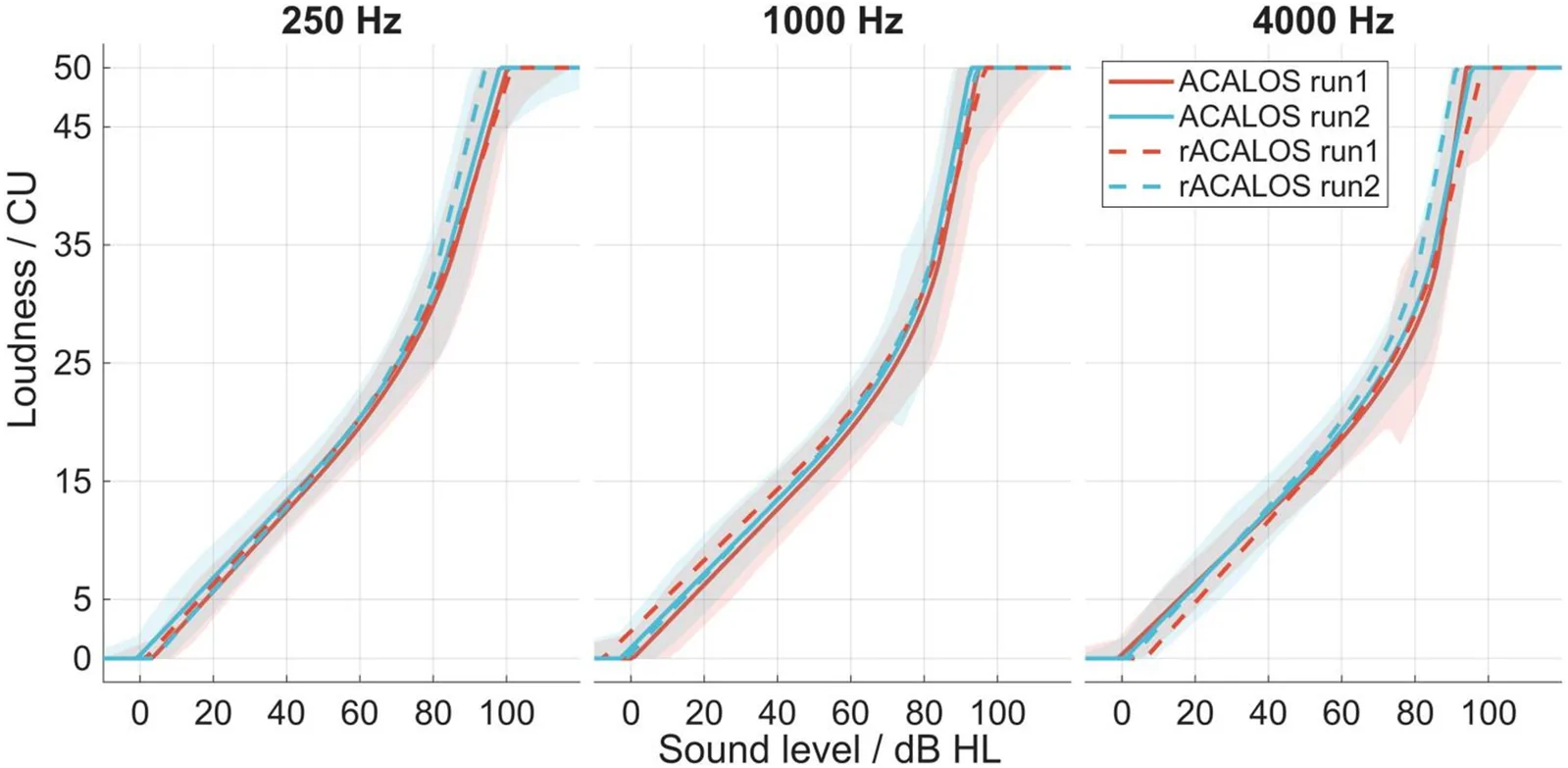
Mean fitted loudness growth functions for ACALOS and rACALOS at 250, 1000, and 4000 Hz. Solid lines indicate ACALOS and dashed lines indicate rACALOS. Shaded regions (Red: Run 1, Blue: Run 2) represent ±1 standard deviation for the ACALOS across participants based on the fitted loudness functions.

## Appendix

### Appendix 1. (Agreement analysis using Bland–Altman plot)

Agreement was further assessed using Bland–Altman analysis. The agreement between two rACALOS runs is shown in Figure A1, while the agreement between in-lab ACALOS and remote rACALOS is presented in Figure A2.

For the test–retest comparison (Figure A1), the mean difference was 0.38 dB, with limits of agreement ranging from −20.3 to 21.9 dB, indicating good repeatability of the rACALOS procedure. For the comparison between in-lab ACALOS and remote rACALOS (Figure A2), the mean difference was −3.14 dB, with limits of agreement ranging from −26.3 to 20.0 dB, suggesting reasonable agreement between the two methods. These results are comparable to those reported by Fultz et al. (2020); (see Figures 5 and 6). Overall, the limits of agreement observed for rACALOS are considered to be clinically acceptable in the context of remote loudness scaling measurements.

### Appendix 2. (Variability of the loudness growth functions using the ACALOS)

Figure S3 illustrates the variability of the ACALOS loudness growth functions. The substantial overlap of the ±1 standard deviation bands suggests similar variability between Runs 1 and 2.

### Glossary

#### Abbreviation Meaning

ACALOS: Adaptive categorical loudness scaling
ANOVA: Analysis of variance
B&K: Brüel & Kjaer
BTUX: Fitting method for loudness function in ACALOS
CLS: Categorical loudness scaling
CU: Categorical units
FL: Fixed-level procedure
GRaBr: Graded response bracketing
HI: Hearing impaired
HTL: Hearing threshold level (at 2.5 CU on the loudness function)
ICC: Intraclass cross-correlation
IQR: Interquartile ranges
MEL-Med: Maximum expected information-median
MEL-ML: Maximum expected information-maximum likelihood
MIQR: Mean interquartile range
MPANLs: Maximum permissible ambient noise levels
MSD: Mean signed difference
NCE: Noise reduction earphones
NH: Normal hearing
PTA: Pure-tone average
qCLS: Quick categorical loudness scaling
rACALOS: Reinforced adaptive categorical loudness scaling
RMSE: Root mean squared error
SA: Slope-adaptive procedure
SIUD: Single interval up and down
SPL: Sound pressure level
