# Supplementary material for: Test–Retest Reliability and Preliminary Validation of Smartphone-Based Threshold and Categorical loudness Measures in Home Settings
Source: Trends Hear. 2026 Aug 20;30:23312165261471286. doi: 10.1177/23312165261471286 (PMC13494286; doi:10.1177/23312165261471286)
Supplement: Supplementary file 1 [file sj-pdf-1-tia-10.1177_23312165261471286.pdf]

Table S1. Validation of the home-based rACALOS procedure for loudness measurements against the in-lab ACALOS reference. Differences in sound levels corresponding to each categorical unit (CU) are shown. Spearman correlation coefficients (R), root-mean-square error (RMSE), and bias are reported for each categorical unit and for the overall average.

loudness	R (“spearman”)	RMSE (dB)	Bias (dB)
not heard (0 CU)	0.46	16.6	1.8
very soft (5 CU)	0.44	12.3	-6.1
15 CU	0.33	14.1	-3.1
25 CU	0.20	11.0	-3.8
35 CU	0.57	11.4	-3.5
45 CU	0.64	7.8	-4.7
50 CU	0.62	7.0	-5.2
