# Supplementary material for: Test–Retest Reliability and Preliminary Validation of Smartphone-Based Threshold and Categorical loudness Measures in Home Settings
Source: Trends Hear. 2026 Aug 20;30:23312165261471286. doi: 10.1177/23312165261471286 (PMC13494286; doi:10.1177/23312165261471286)
Supplement: Supplementary file 2 [file sj-pdf-2-tia-10.1177_23312165261471286.pdf]

Table S2. Test-retest reliability of ACALOS and rACALOS at three frequencies (i.e., 0.25, 1, and 4 kHz) between the test and retest measurements. Across-run bias and within-run variability are calculated as mean signed difference (MSD) and mean interquartile range (MIQR), respectively. The two metrics as a function of loudness (11 categories) are reported.

	ACALOS						rACALOS					
	250Hz		1000Hz		4000Hz		250Hz		1000Hz		4000Hz	
Loudness (CU)	MSD/MIQR		MSD/MIQR		MSD/MIQR		MSD/MIQR		MSD/MIQR		MSD/MIQR	
0 (not heard)	4.3	9.0	1.0	5.7	5.9	3.8	0.1	4.5	-5.3	4.9	11.0	5.0
5 (very soft)	3.3	10.8	-3.3	10.4	0.2	9.5	-0.9	5.1	-3.5	7.7	6.8	6.7
10	-1.2	11.7	1.7	14.9	-1.0	12.4	-3.8	11.0	0.4	16.6	-2.6	12.5
15 (soft)	-0.4	12.7	3.1	13.9	6.4	13.7	-2.1	13.8	1.0	17.1	0.6	18.4
20	2.5	6.7	5.0	4.6	4.7	9.8	2.9	11.6	2.8	7.7	2.7	15.7
25 (medium)	2.7	7.6	-1.9	5.8	3.7	3.5	2.4	9.9	0.4	11.0	0.1	5.2
30	4.0	3.4	1.2	4.2	1.5	3.2	1.0	2.9	-2.9	0.6	5.8	3.3
35 (loud)	0.9	5.9	2.5	4.1	-0.3	4.4	-0.6	5.1	-2.0	2.9	3.6	4.5
40	2.2	5.8	2.7	1.5	1.2	0.0	4.5	0.2	1.1	3.8	-1.1	0.0
45 (very loud)	9.0	3.2	-0.3	3.6	-2.5	2.1	1.1	2.3	-0.2	4.3	0.4	2.0
50 (too loud)	3.5	5.4	1.4	1.7	0.1	2.3	0.0	0.0	1.5	0.0	1.1	1.8
